# Circulating miR-150 and miR-342 in plasma are novel potential biomarkers for acute myeloid leukemia

**DOI:** 10.1186/1479-5876-11-31

**Published:** 2013-02-07

**Authors:** Hussein Fayyad-Kazan, Nizar Bitar, Mehdi Najar, Philippe Lewalle, Mohammad Fayyad-Kazan, Rabih Badran, Eva Hamade, Ahmad Daher, Nader Hussein, Rim ELDirani, Fadwa Berri, Luc Vanhamme, Arsène Burny, Philippe Martiat, Redouane Rouas, Bassam Badran

**Affiliations:** 1 Laboratory of Experimental Hematology, Institut Jules Bordet, Université Libre de Bruxelles, 121, Boulevard de Waterloo, Bruxelles 1000, Belgium; 2 Department of Biochemistry, Laboratory of Immunology, EDST-PRASE, Lebanese University, Faculty of Sciences, Hadath-Beirut, Lebanon; 3 Laboratory of Clinical Cell Therapy (LTCC), Hôpital Erasme, Université libre de Bruxelles (ULB), 808, Route de Lennik, Brussels, 1070, Belgium; 4 Laboratory of Membrane Transport Biology, IBMM (Institute for Molecular Biology and Medicine), Université Libre de Bruxelles, 12 Rue des Professeurs Jeener et Brachet, Gosselies, 6041, Belgium; 5 Laboratory of Molecular Parasitology and Laboratory of Molecular Biology of Ectoparasites, IBMM (Institute for Molecular Biology and Medicine), Université Libre de Bruxelles, 12 Rue des Professeurs Jeener et Brachet, Gosselies, 6041, Belgium

**Keywords:** AML, Micro-RNA, Plasma, Biomarker

## Abstract

**Background:**

MicroRNAs (miRNAs) are small (19-22-nt) single-stranded noncoding RNA molecules whose deregulation of expression can contribute to human disease including the multistep processes of carcinogenesis in human. Circulating miRNAs are emerging biomarkers in many diseases and cancers such as type 2 diabetes, pulmonary disease, colorectal cancer, and gastric cancer among others; however, defining a plasma miRNA signature in acute myeloblastic leukemia (AML) that could serve as a biomarker for diagnosis or in the follow-up has not been done yet.

**Methods:**

TaqMan miRNA microarray was performed to identify deregulated miRNAs in the plasma of AML patients. Quantitative real-time RT-PCR was used to validate the results. Receiver-operator characteristic (ROC) curve analysis was conducted to evaluate the diagnostic accuracy of the highly and significantly identified deregulated miRNA(s) as potential candidate biomarker(s).

**Results:**

The plasma expression level of let-7d, miR-150, miR-339, and miR-342 was down-regulated whilst that of let-7b, and miR-523 was up-regulated in the AML group at diagnosis compared to healthy controls. ROC curve analyses revealed an AUC (the areas under the ROC curve) of 0.835 (95% CI: 0.7119– 0.9581; P<0.0001) and 0.8125 (95% CI: 0.6796–0.9454; P=0.0005) for miR-150, and miR-342 respectively. Combined ROC analyses using these 2 miRNAs revealed an elevated AUC of 0.86 (95% CI: 0.7819–0.94; P<0.0001) indicating the additive effect in the diagnostic value of these 2 miRNAs. QRT-PCR results showed that the expression level of these two miRs in complete remission AML patients resembled that of healthy controls.

**Conclusions:**

Our findings indicated that plasma miR-150 and miR-342 are novel important promising biomarkers in the diagnosis of AML. These novel and promising markers warrant validation in larger prospective studies.

## Background

Acute myeloid leukemia (AML) is a clonal disorder caused by malignant transformation of a bone marrow-derived progenitor cell, which demonstrates an enhanced proliferation as well as aberrant differentiation resulting in hematopoietic insufficiency (i.e. granulocytopenia, thrombocytopenia or anaemia) [1,2]. AML is the most common type of acute leukemia occurring in adults, with approximately 11,900 individuals diagnosed annually in the United States only [3]. Adults over age 60 comprise more than two-thirds of this group [2], with a median age of onset of about 65 [3]. The incidence of AML rises significantly with age, with 4 cases per 100,000 people annually in the sixth decade of life, to over 20 cases per 100,000 in the ninth decade of life [4]. An increased incidence of AML is seen in patients with disorders associated with excessive chromatin fragility such as Bloom syndrome, Fanconi anemia, Kostmann syndrome, Wiskott-Aldrich syndrome or ataxia telangiectasia syndrome. Other syndromes, such as Down (trisomy 21), Klinefelter (XXY and variants), and Patau (trisomy 13), have also been associated with a higher incidence of AML [5-8]. In fact, AML patients in remission frequently relapse due to the addition of new mutations [9].

In general, adults with AML show a variety of symptoms including fatigue, bruising or bleeding, fever, and infection, reflecting a state of bone marrow failure [10]. Physical findings other than bleeding and infection may include organomegaly, lymphadenopathy, sternal tenderness, retinal hemorrhages, and infiltration of gingivae, skin, soft tissues, or meninges (more common with monocytic variants M4 or M5) [10]. The diagnosis of AML is often demonstrated by an increased number of myeloblasts in the bone marrow or peripheral blood. According to the WHO criteria, acute leukemia is diagnosed when a 200-cell differential reveals the presence of 20% or more myeloblasts in a marrow aspirate or in blood [11].

MicroRNAs (miRNAs) are small (19-22-nt) single-stranded noncoding RNA molecules that are derived from hairpin-structured precursors [12]. These microRNAs function by directly binding to their potential target site in the 3^′^ untranslated region (3^′^UTRs) of specific target mRNA, leading to the repression of mRNA translation or the degradation of target mRNAs. Small non-coding RNAs were also recently implicated in control of DNA-damage response [13]. Currently, there are 2042 mature human miRNA sequences listed in the miRNA registry (Sanger miRBase release 19; http://www.mirbase.org/). Over recent years, many miRNAs have been investigated in various human cancers [14,15]. The deregulation of expression of microRNAs has been shown to contribute to the multistep processes of carcinogenesis in human by modification either of oncogenic or suppressor gene function [16,17]. Nowadays, microRNA expression patterns are known to characterize the developmental origins of tumors more effectively than mRNA expression signatures and thus can be a useful tool for the diagnosis and prognosis of human cancer [18]. The search for non-invasive tools for the diagnosis and management of cancer that can greatly reduce its worldwide health burden has long been a goal of cancer research [19]. Recently, it has been reported that microRNAs are circulating in serum/plasma [20,21] and tumor-derived microRNAs such as miR-155, miR-21, miR-15b, miR-16 and miR-24 have been detected in the plasma and sera of tumor-bearing patients [22,23]. These circulating microRNAs can be considered as a new class of effective biomarkers where their abundance profile might reflect physiological and/or pathological conditions. Accordingly, several subsequent studies have proven that miRNAs can serve as potential biomarkers for various diseases including cancer [20,24,25].

A previous report [26] had shown that down-regulation of miR-92 is a novel marker for acute leukemia patients (AML, ALL). In our study, we investigated the profile of circulating microRNAs in the plasma of acute myeloid leukemia patients compared with healthy individuals. Our results have revealed the presence of two microRNAs (miR-150, miR-342) whose levels were very significantly downregulated in the plasma of AML patients at diagnosis compared to healthy controls. The combination of their decrease is of utmost significance using ROC curve analysis. Investigation of the expression level of these two microRNAs in complete remission (CR) AML patients by qRT-PCR revealed a similar expression level as that of healthy controls. Thus, besides miR-92, these two microRNAs are novel candidate biomarkers of acute myeloid leukemia and potential predictors of relapse.

## Methods

### Patients

Patients used in this study had a newly diagnosed AML in addition to being in complete remission as determined by blood test. A total of 20 patients at the time of diagnosis in addition to other 20 patients in complete remission provided blood samples. Healthy subjects were collected as negative controls. None of these controls had previously been diagnosed with any type of malignancy or other benign disease. Informed consent, approved by the Clinical Research Ethics Committee of Jules Bordet Institute, was obtained from each participant. Details of clinical data are provided in Table 1.

**Table 1:** Summary of clinical details of AML patients and healthy controls used for analysis

**Sex**	
**Men**	**13**
**Women**	**7**
**Total**	**20**
**Control**	**20**
**French-American—British classification**	
**AML M0**	**3**
**AML M1**	**3**
**AML M2**	**2**
**AML M3**	**2**
**AML M4**	**2**
**AML M4E0**	**2**
**AML M5**	**2**
**AML M6**	**2**
**AML M7**	**2**

The classification was done according to the French-American-British system [27].

M0= Undifferentiated acute myeloblastic leukemia; M1= Acute myeloblastic leukemia with minimal maturation; M2= Acute myeloblastic leukemia with maturation; M3=Acute promyelocytic leukemia (APL); M4= Acute myelomonocytic leukemia; M4E0=Acute myelomonocytic leukemia with eosinophilia; M5= Acute monocytic leukemia; M6= Acute erythroid leukemia; M7= Acute megakaryoblastic leukemia.

### Plasma sampling and RNA extraction

At presentation, blood samples for miRNA detection were collected in EDTA-K2 tubes and processed within 1 hour of collection. Blood samples were centrifuged at 1,200 g for 10 min at 4°C to spin down the blood cells, and the supernatant was transferred into microcentrifuge tubes, followed by a second centrifugation at 12,000 g for 10 min at 4°C. The supernatant was transferred to RNase/DNase-free tubes and stored at −80°C. Total RNA was isolated from the plasma using a mirVana PARIS isolation kit (Ambion, Austin, Texas) according to the manufacturer’s instructions for plasma samples. Briefly, 400 μL of human plasma was used to extract total RNA. Each sample was eluted in 100 μL of RNAse-free water and was concentrated to a final volume of 20 μL by using Eppendorf Concentrator Plus 5301 (Eppendorf, Germany).

RNA sample concentration was quantified by NanoDrop ND-1000 (Nanodrop, USA). All RNA samples were analyzed for miR-16 expression, a stable endogenous reference miRNA, to assess an approximate yield of RNA extraction and to ensure that comparable amounts of starting material were used in each reverse transcription reaction [22,28-31].

### MiRNA expression profile

In our study, a three-step procedure was performed to profile the miRNAs in the plasma samples. First, for cDNA synthesis from the miRNAs, 30 ng of total RNA was subjected to RT (reverse transcription) using a TaqMan^® ^microRNA Reverse Transcription Kit (#4366596; Applied Biosystems) and Megaplex RT primers (Human Pool A, #4399966; Applied Biosystems) following the manufacturer’s protocol, allowing simultaneous reverse transcription of 380 mature human miRNAs to generate a miRNA cDNA library corresponding to each plasma sample. RT was performed on a Mastercycler Epgradient thermocycler (Eppendorf) with the following cycling conditions: 40 cycles at 16°C for 2 min, 42°C for 1 min and 50°C for 1 s followed by a final step of 80°C for 5 min to inactivate reverse transcriptase. Thereafter, to generate enough miRNA cDNA template for the following real-time PCR, the cDNA libraries were pre-amplified using Megaplex PreAmp primer (Humam Pool A, #4399233; Applied Biosystems) and PreAmp Master Mix (#4384266; AppliedBiosystems) following the manufacturer’s instructions. The PreAmp primer pool used here consisted of forward primers specific for each of the 380 human miRNAs and a universal reverse primer. The pre-amplification cycling conditions were as follows: 95°C for 10 min, 55°C for 2 min, 72°C for 2 min followed by 12 cycles at 95°C for 30 s and 60°C for 4 min; the samples were then held at 99.9°C for 10 min. After the preamplification step, the products were diluted with RNase-free water, combined with TaqMan gene expression Master Mix and then loaded into TaqMan Human MicroRNA Array A (#4398965; Applied Biosystems), which is a 384-well formatted plate and real-time PCR-based microfluidic card with embedded TaqMan primers and probes in each well for the 380 different mature human miRNAs; MiR-16 transcript was used as a normalization signal.

Real-time PCR was performed on an ABI PRISM 7900HT sequence detection system (Applied Biosystems) with the following cycling conditions: 50°C for 2 min, 94.5°C for 10 min followed by 40 cycles at 95°C for 30 s and 59.7°C for 1 min. The Ct (cycle threshold) was automatically given by SDS 2.3 software (Applied Biosystems) and is defined as the fractional cycle number at which the fluorescence passes the fixed threshold of 0.2. MiR-16 embedded in the TaqMan Human MicroRNA Arrays was used as an endogenous control. The relative expression levels of miRNAs were calculated using the comparative ΔΔCt method as described previously [32,33]. The fold changes in miRNAs were calculated by the equation 2^−ΔΔCt^.

### Taqman miRNA assay for individual miRNAs

Gene-specific reverse transcription was performed for each miR using 10 ng of purified total RNA, 100 mM dNTPs, 50 U MultiScribe reverse transcriptase, 20 U RNase inhibitor, and 50 nM of gene-specific RT primer samples using the TaqMan MicroRNA Reverse Transcription kit (Applied Biosystems, Gent, Belgium). 15 μl reactions were incubated for 30 min at 16°C, 30 min at 42°C, and 5 min at 85°C to inactivate the reverse transcriptase. Real time RT-PCR reactions (5 μl of RT product, 10 μl TaqMan 2x Universal PCR master Mix, (Applied Biosystems, Gent, Belgium), and 1 μl TaqMan MicroRNA Assay Mix containing PCR primers and TaqMan probes) were carried out on ABI Prism 7900HT Sequence Detection System (Applied Biosystems, Gent, Belgium) at 95°C for 10 min followed by 40 cycles at 95°C for 15 s and 60°C for 1 min. The qRT-PCR reactions were performed in triplicate, and the signal was collected at the end of every cycle. Due to a lack of generally accepted standards, all qRT-PCR data on single miRNA expression were analyzed as unadjusted Ct values and standardized to miR-16. To validate miR-16 as a stable internal reference, its stability during extraction was compared to that of synthetic cel-miR-39, a miRNA of C. elegans that is not present in humans. Twenty-five fmol of synthetic cel-miR-39 were spiked in after adding the Denaturing Solution (provided in the mirVana PARIS isolation kit) to the human plasma samples to avoid degradation by endogenous RNases, and the RNA was extracted. We measured the expression of cel-miR-39, miR-16 and the validated differentially expressed microRNAs in AML patients (diagnosis and complete remission) and healthy controls. Afterwards, the expression of the validated microRNAs in AML patients and healthy controls was compared with miR-16 and cel-miR-39 normalizers, respectively.

### Statistical analysis

Data characterized by a normal distribution, determined using Kolmogorov-Smirnov and Shapiro-Wilk normality tests, were expressed as the mean and standard deviation. Widely presented using the 2^-ΔΔCt^ method, the relative gene expression involves the gene of interest data (Ct gene of interest) relative to an internal control gene (Ct internal control gene), named delta Ct. The calculated delta Ct ± SD for the patients was compared with the delta Ct ± SD (SD stands for the standard deviation of the average delta Ct of the group) for the healthy control group and tested for statistical significance. Sensitivity, specificity, and the area under the curve (AUC) for plasma microRNAs were determined using Receiver Operator Characteristic (ROC) analysis. Data were analyzed using Student’s t test. P-values <0.05 (*), <0.01 (**), and <0.001(***) obtained using t-test were considered statistically significant.

## Results

### Expression profiles of miRNAs in the plasma of AML patients

RNA from twenty independent human AML patients and healthy controls was first studied using the TLDA technique. We could identify several miRs that were statistically differentially expressed between AML and healthy controls (Table 2). These microRNAs were further studied to validate their differential expression by quantitative Real Time PCR (qRT-PCR) and to investigate whether anyone could be used as a candidate biomarker of AML at the diagnosis.

**Table 2:** Circulating plasma microRNA expression levels in AML patients compared to healthy controls

**MicroRNA**	**AML/Healthy control ratio**	**P value**
**Hsa-let-7b**	**6.5**	**0.021**
**Hsa-let-7d**	**0.2**	**0.026**
**Hsa-miR-150**	**0.045**	**0.0026**
**Hsa-miR-335**	**4.5**	**0.041**
**Hsa-miR-339**	**0.4**	**0.031**
**Hsa-miR-342**	**0.07**	**0.0048**
**Hsa-miR-374**	**3.5**	**0.045**
**Hsa-miR-523**	**5**	**0.022**

### Validation of candidate miRNAs

**Figure 1:**
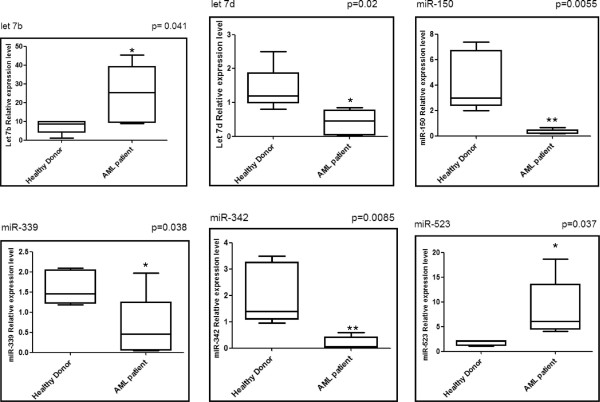
**Relative expression levels of the six differentially expressed miRs. **Six miRNAs are significantly differentially expressed in the plasma of AML patients when compared to healthy controls (n=20). Data obtained by quantitative RT-PCR amplification of miRs are plotted. p-values for each miRNA are shown. Boxes represent SE. Error bars represent SD; pooled data from five independent experiments. *p<0.05, **p<0.01 AML patient versus Healthy controls (Student’s t-test).

**Figure 2:**
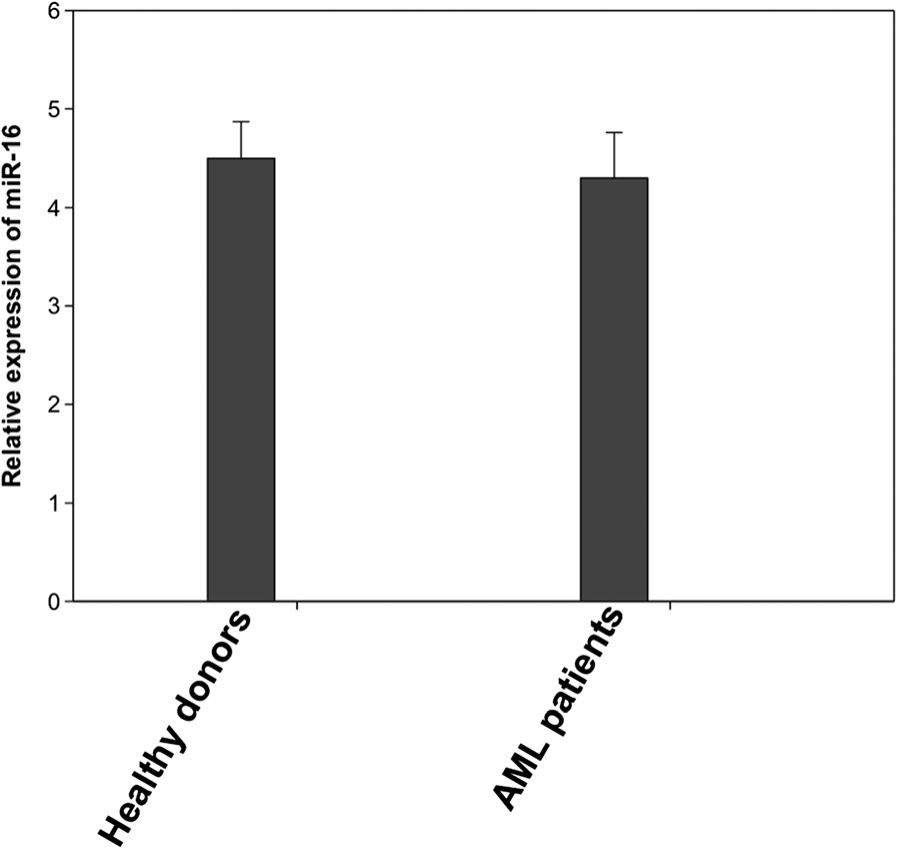
**miR-16 expression level is stable in both healthy controls and AML patients. **MiR-16 expression levels were assessed by qRT-PCR and normalized by cel-miR-39. Shown are the relative levels (mean ± S.D.) of five independent experiments performed on all participants, each done in triplicate.

**Figure 3:**
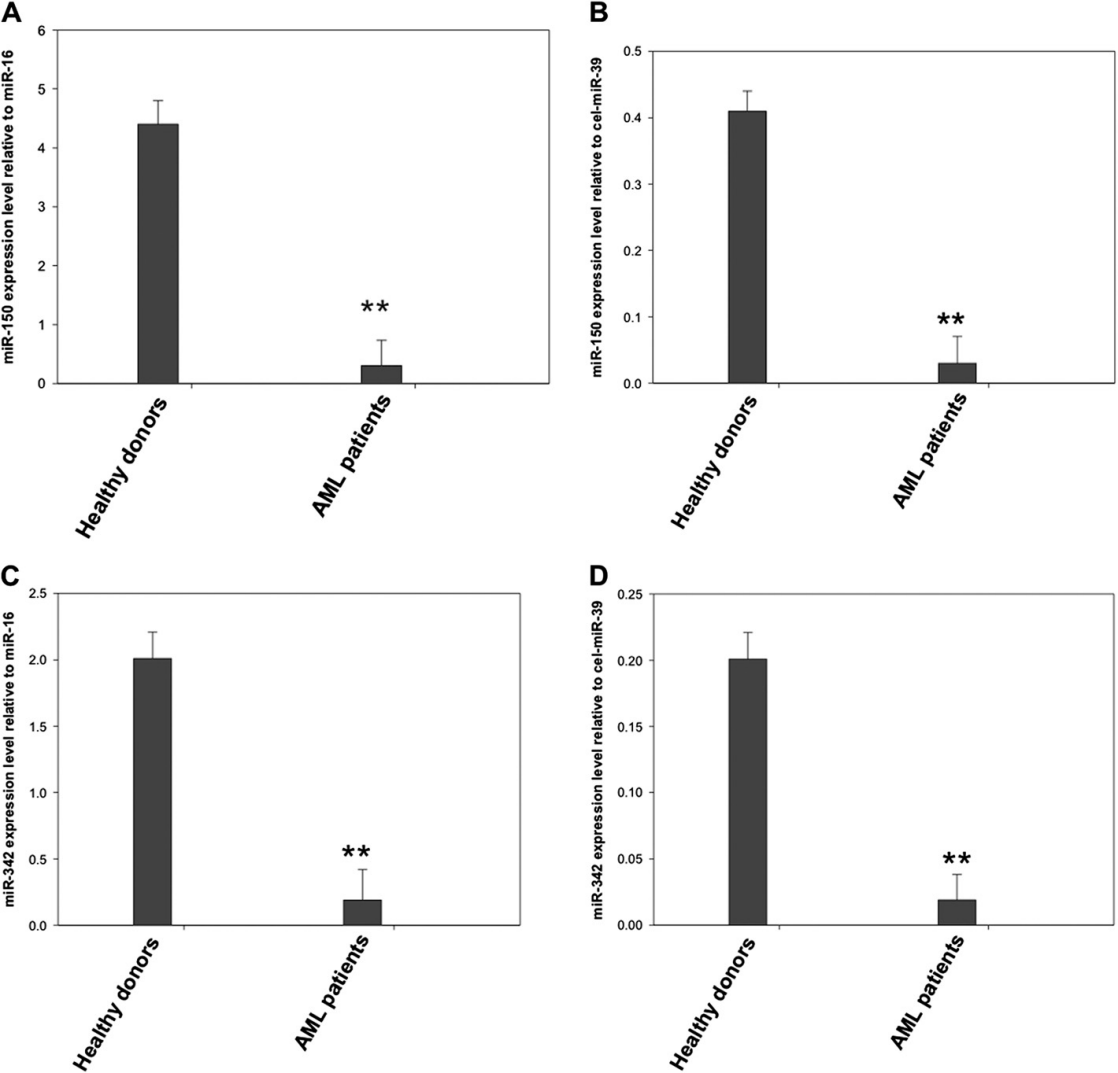
**Relative plasma miR-150 and miR-342 expression levels normalized by cel-miR-39 and hsa-miR-16. **MiR-150 **(A, B) **and miR-342 **(C, D) **expression levels were assessed by qRT-PCR and normalized by cel-miR-39 and hsa-miR-16 respectively in healthy controls and Acute Myeloid Leukemia patients. Shown are the relative levels (mean ± S.D.) of five independent experiments performed on all participants, each done in triplicate. Statistical significance was determined by Student’s t test and is denoted as follows: ** *p <*0.01 *versus *healthy donors.

Differential miR expressions were validated by real-time PCR in all samples. The change in candidate miRNAs for the AML patients versus the healthy controls is shown in Figure 1. These data have been normalized by the expression level of miR-16, a widely used endogenous reference miRNA that was also confirmed to be unchanged in our experiments (TLDA cards). In addition, as cel-miR-39, miR-16 is stable (Figure 2). Moreover, we compared the difference in let-7b, let-7d, miR-150, miR-339, miR-342, and miR-523 expression between AML patients and healthy controls and obtained the same differences in their expression regardless of whether cel-miR-39 or miR-16 was used as the normalizer (Figure 3), which further supports that miR-16 is a stable reference in this study.

**Figure 4:**
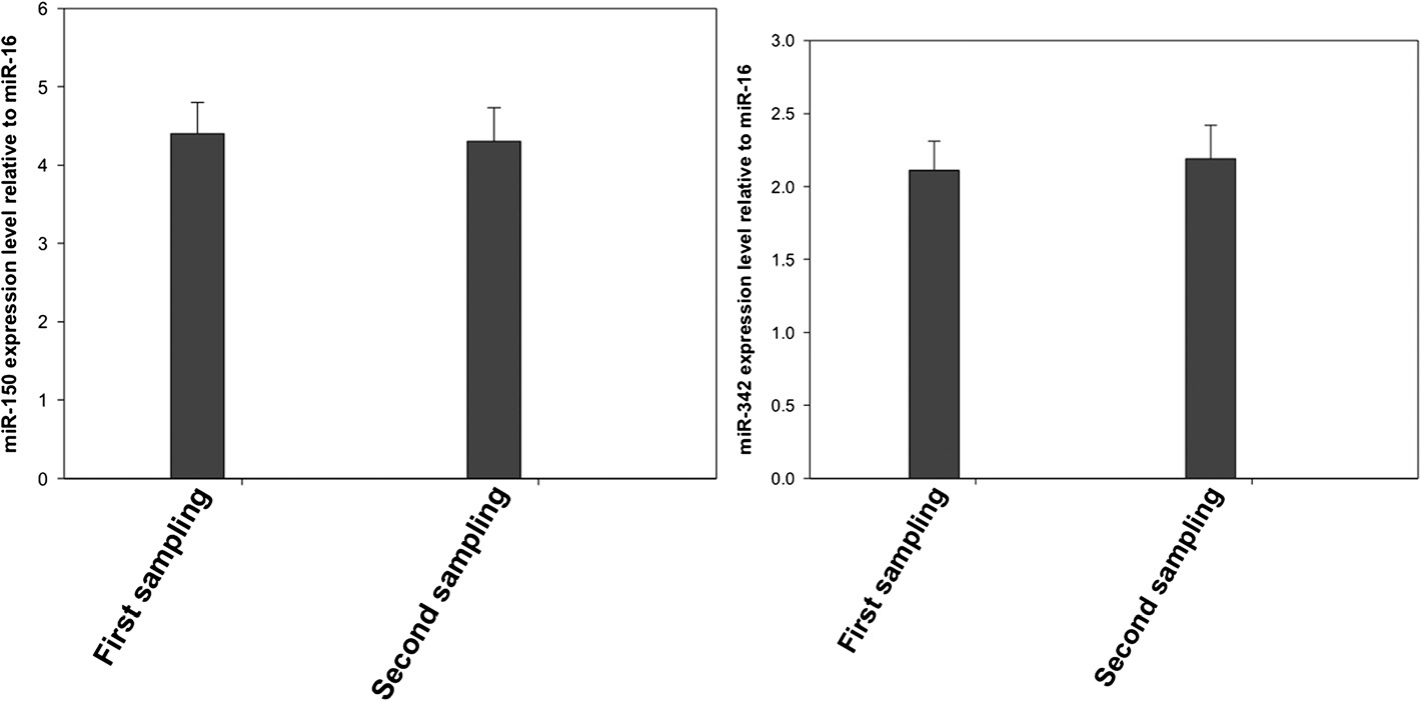
**miR-150 and miR-342 expression levels do not vary between the first and second sampling. **Plasma miR-150 and miR-342 expression levels in the first sampling and second sampling were assessed by qRT-PCR. The expression level of these two microRNAs was normalized to miR-16. Shown are the relative levels (mean ± S.D.) of five independent experiments performed on all participants, each done in triplicate.

The plasma expression level of let-7d, miR-150, miR-339, and miR-342 was down-regulated whilst that of let-7b, and miR-523 was up-regulated in the AML group compared to healthy controls (Figure 1). To confirm that the assay is reproducible, we also analyzed expression levels in the plasma (second sampling) collected 1 h after the first sampling of the plasma of three AML patients. No significant difference in the levels of the above mentioned microRNAs was found between the first sampling and the second sampling, which suggests that the assay is reproducible (Figure 4).

### Diagnostic accuracy of plasma miR-150 and miR-342 in AML

**Figure 5:**
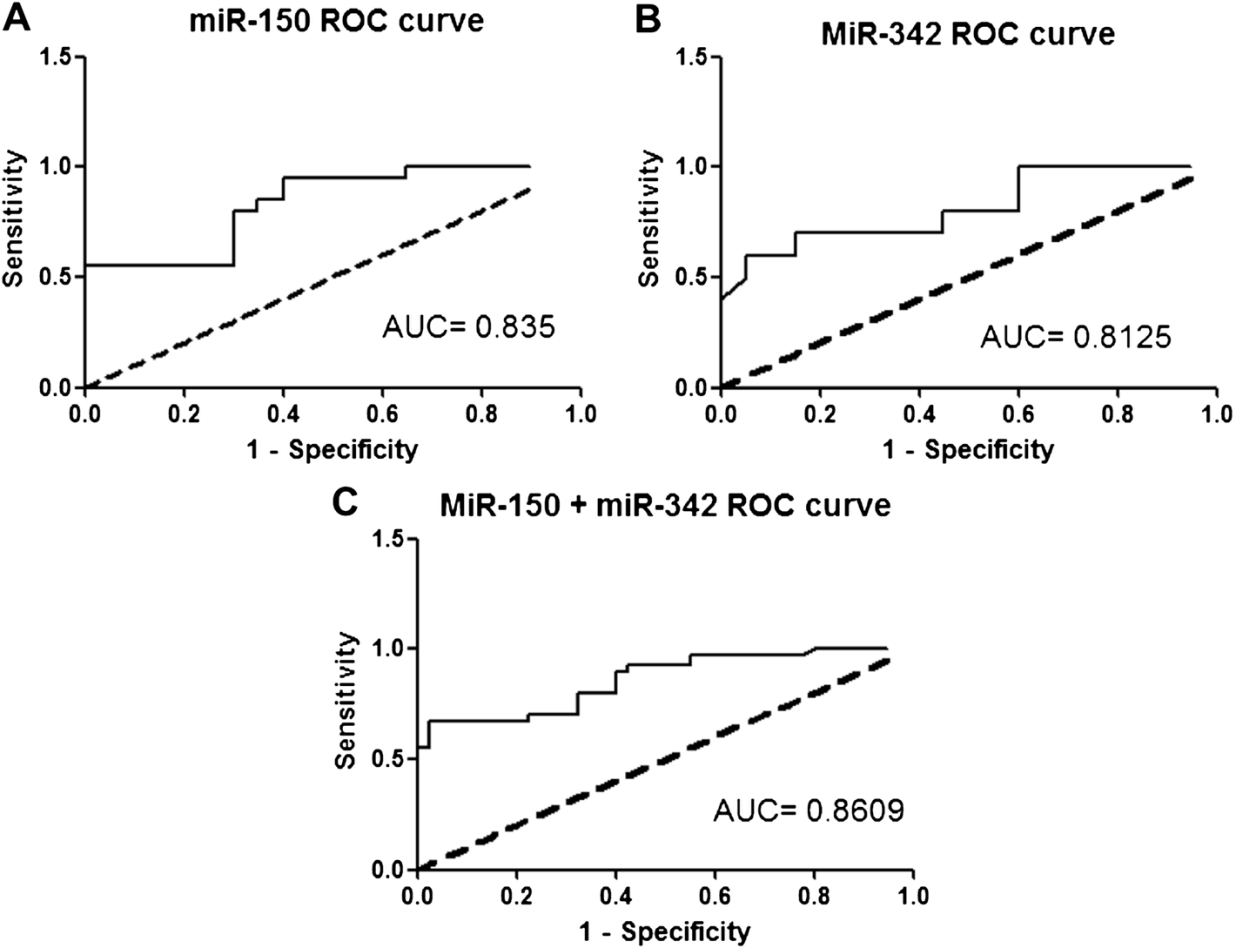
**Receiver operating characteristics (ROC) curve analysis using plasma miR-150 and miR-342 for discriminating AML patients. **Plasma miR-150 yielded an AUC (the areas under the ROC curve) of 0.835 (95% CI: 0.7119– 0.9581; P<0.0001) with 80% sensitivity and 70% specificity in discriminating AML **(A)**, and plasma miR-342 yielded AUC of 0.8125 (95% CI: 0.6796–0.9454; P=0.0005) with 70% sensitivity and 85% specificity **(B) **in discriminating AML. Elevated ROC analysis revealed an elevated AUC of 0.860 (95% CI: 0.7819–0.94; P<0.0001) with 73% sensitivity and 78% specificity in discriminating AML **(C)**.

The ROC curve analysis was used to analyze the diagnostic accuracy of plasma miR-150 and miR-342. ROC curve analyses revealed that both plasma miR-150 and miR-342 could serve as valuable biomarkers for differentiating AML from controls with an AUC (the areas under the ROC curve) of 0.835 (95% CI: 0.7119– 0.9581; P<0.0001) and 0.8125 (95% CI: 0.6796–0.9454; P=0.0005), respectively (Figures 5A and 5B).

At the cut-off value less than 2.79 for miR-150, the sensitivity and the specificity were 80% and 70%, respectively. At the cut-off value less than 1.146 for miR-342, the sensitivity and the specificity were 70% and 85%, respectively. Combination ROC analyses resulted in an increased AUC of 0.86 (95% CI: 0.7819–0.94; P<0.0001) with 73.0% sensitivity and 78% specificity indicating the additive effect in the diagnostic value of these 2 miRNAs (Figure 5C).

### MiR-150 and miR-342 in CR AML patients showed an expression level similar to that of healthy controls

**Figure 6:**
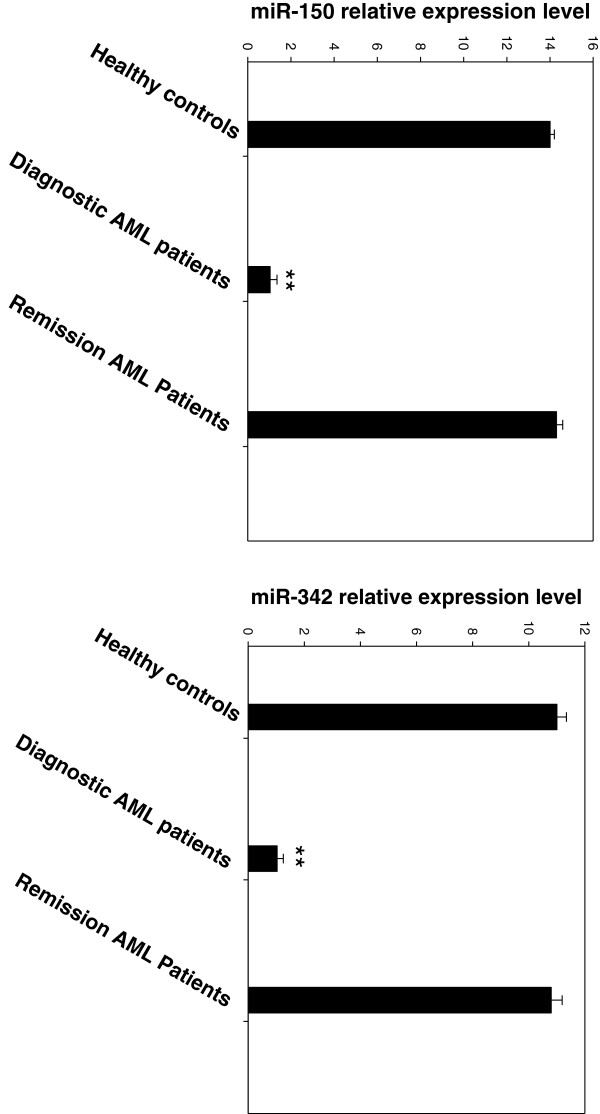
**miR-150 and miR-342 expression levels in Remission AML patients resembles that of healthy controls. **Plasma miR-150 and miR-342 expression levels in remission AML patients and healthy controls were assessed by qRT-PCR. The expression level of these two microRNAs was normalized to miR-16. Shown are the relative levels (mean ± S.D.) of five independent experiments performed on all controls, each done in triplicate. Statistical significance was determined by Student’s t test and is denoted as follows: ** *p <*0.01 *versus *healthy donors.

ROC curve analyses revealed that plasma miR-150 and miR-342 could serve as valuable biomarkers for differentiating AML from controls. Thus, in order to confirm that, we assessed the expression level of these two microRNAs in CR AML patients, compared to AML patients at diagnosis and healthy controls, using qRT-PCR. Results showed that the plasma expression level of miR-150 and miR-342 was similar to that of healthy controls while it was still upregulated compared to AML patients at diagnosis (Figure 6) thus making these two microRNAs as additional novel biomarkers for AML.

## Discussion

In this study, we identified several microRNAs in the plasma of AML patients at diagnosis that were differentially expressed compared to healthy controls. Among these miRs, two were upregulated (Let-7b, miR-523) and four were downregulated (let-7d, miR-150, miR-339, and miR-342). Importantly, the expression level of these microRNAs didn’t show any significant difference between the male and female donors implicated in this study as revealed by student’s t- test statistical analysis. The latter was performed based on the Kolmogorov-Smirnov and Shapiro-Wilk normality analysis tests. Among these microRNAs, miR-150, and miR-342 were very significantly downregulated in the plasma of AML patients as confirmed using ROC curve analysis (AUC of 0.835 and 0.8125) that revealed that miR-150, and miR-342 were promising candidate biomarkers for AML at diagnosis. These data suggest that microRNA expression signature in plasma can serve as a valuable diagnostic and potential prognostic marker for human AML. In that respect, the rebound in CR AML patients of miR-150 and miR-342 at the healthy controls’ levels is of particular significance in the perspective of their use as diagnostic and prognostic factors. Recent studies have revealed that miRNAs are potential diagnostic biomarkers and prognostic factors in cancers [34,35]. Mitchell et al. [23] were the first to identify the presence of circulating tumor-associated miRNAs in plasma and to show that circulating microRNAs may have an important value for cancer diagnosis. Another group [24] also determined that circulating miRNA profiles in patients with lung cancer, colorectal cancer (CRC), and diabetes had a prognostic significance. Although the clinical significance of these observations has not been elucidated in detail, those findings demonstrated that circulating miRNAs could be non-invasive diagnostic or prognostic markers for cancer and in this case for AML. A recent study [35] showed that miR-150 was downregulated in sepsis, both in granulocytes and monocytes and inversely correlated with the severity of the disease. Interestingly, plasma levels of tumor necrosis factor alpha, interleukin-10, and interleukin-18, which all have a complementary sequence to miR-150, were negatively correlated with the plasma levels of miR-150. This could impact on the immune system response to leukemia. On the other hand, an important report [36] related to miR-342 demonstrated the importance of its decreased plasma level in breast cancer, particularly in resistance to certain agents.

From a technical point of view, normalization is a key step for the accurate quantification of RNA levels with qRT-PCR. In our study, miR-16 was used as an internal control for plasma miRNA quantification as is the case of other studies carried out on different tumors, including CRC [25] breast cancer [37] ovarian cancer [38] where miR-16 was present in plasma/serum at similar levels across normal controls and patients. In addition to the normalization of qRT-PCR data, appropriate control is also a key issue for diagnostic studies. It is therefore crucial to ensure that the control group is free of any disease, even benign, which is the case in this study. Larger sample size may also be helpful to eliminate potential sampling error.

Exosomes are small (50–90 nm) membrane vesicles of endocytic origin that are released into the extracellular environment on fusion of multivesicular bodies (MVB) with the plasma membrane [39]. Many cells including reticulocytes [40], dendritic cells [41], B cells [42], T cells [43], mast cells [44], epithelial cells [45] and tumor cells [46] have the capacity to release exosomes. Exosomes’ content, notably microRNAs known to exist in a form that is resistant to plasmatic RNase activity [23], can be delivered to another cell and function in a new location [47]. These studies suggest that microRNAs are packaged inside exosomes that are secreted from cells. Thus, it might be possible that cancer cells specifically take in the exosome that contains miR-150 and miR-342 and as a result, miR-150 and miR-342 decrease in the plasma.

In the study quoted above [35], the authors determined that the level of miR-150 was independent from the number of white blood cells (WBC), but decreased both in these normal WBCs and in the plasma. However, the mechanism was not elucidated. Thus, an alternative explanation could be that leukemic process creates a decrease in the normal WBC compartment and consequently in the plasma. This is of course purely speculative, and further experiments should address the mechanisms involved.

In summary, we have shown that the expression level of miR-150 and miR-342 in plasma is associated with diagnosis of AML in human. Present knowledge does not involve circulating miR-150 and miR-342 as references in other human cancers [48,49].

## Conclusions

In our study, we identified 8 miRNAs differentially expressed between plasma of AML patients and healthy controls by TLDA. Among those eight microRNAs, seven were confimed by qRT-PCR; let-7b and miR-523 were upregulated whilst others were downregulated including the two highly significant microRNAs, miR-150, and miR-342. Combination of these miRs has diagnostic value enabling identification of AML with the sensitivity of 73%, specificity 78% and AUC = 0.86. The diagnostic value of these two microRNAs was confirmed where the expression level of these two microRNAs in AML patients at remission resembled that of healthy controls. Thus, we believe, that circulating miR-150 and −342 in plamsa are novel biomarkers in AML.

## Competing interests

The authors declare that they have no competing interests.

## Authors’ contributions

HFK, NB, PM, RR, and BB designed the study. NB, PL, PM, and BB were responsible for recruiting patients for the study. HFK, EH, NH, FB, AD, and RE generated the data. HFK, RB, MFK, LV, AD, PM, RR, and BB analyzed the data. All the authors contributed to drafting and reviewing the manuscript, and all the authors read and approved the final manuscript.

## Authors’ information

Rouas Redouane and Badran Bassam Joint senior co-authors.
